# The time is ripe for eQTLs: Transcriptomic identification of a tomato fruit ripening regulator

**DOI:** 10.1093/plphys/kiac287

**Published:** 2022-06-15

**Authors:** Amy Lanctot

**Affiliations:** Cold Spring Harbor Laboratory, Howard Hughes Medical Institute, Cold Spring Harbor, New York 11724, USA

Fruit ripening is a key developmental step in plant reproductive growth, as seeds mature within the ripening fruit and the fruit itself promotes seed dispersal. Ripening is a complex process requiring the coordination of multiple genetic pathways, including ethylene biosynthesis, cell wall remodeling, carotenoid accumulation, and the production of secondary metabolites (Shinozaki et al., 2018). These pathways are controlled by master regulators, such as the transcription factors RIPENING INHIBITOR (RIN) and NON-RIPENING (NOR) (Tigchelaar, 1973; Vrebalov et al., 2002). Due to its agricultural importance, fruit ripening is an area of active research, and spatially and temporally refined transcriptomic approaches are elucidating the complexities of this essential developmental transition.

In this issue of *Plant Physiology*, Zhu et al. (2022) compare the distinct ripening processes in tomato (*Solanum lycopersicum*) and its wild relative *Solanum pennellii*. Tomato characteristically has red, fleshy fruit, while *S. pennellii* has much smaller, hard, green fruit (Eshed and Zamir, 1995). The coding sequences of many known ripening regulators are identical between these two species, suggesting that these phenotypic differences arise from expression-level differences rather than changes in protein structure.

**Figure 1:**
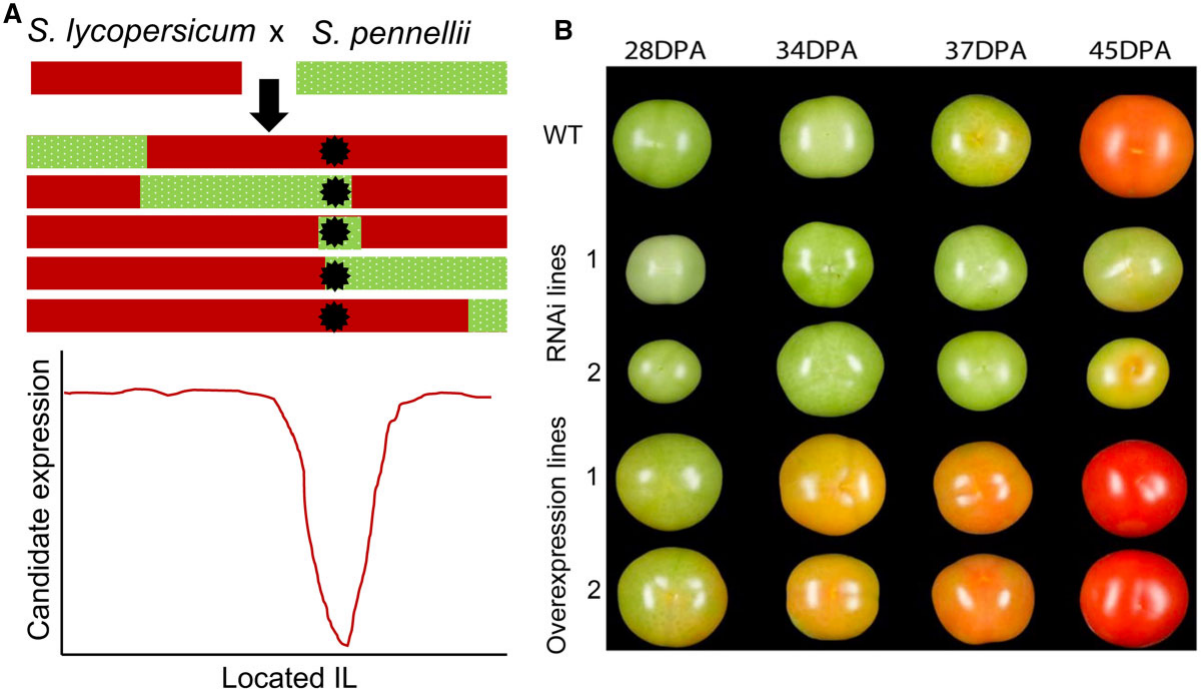
eQTL analysis of an introgressed population of tomato with its wild relative *S. pennellii* identifies a fruit ripening regulator. A, Schematic of eQTL approach. The expression of differentially expressed genes in tomato fruit is assessed in the fruit of ILs. Genes whose expression sharply decreases in ILs where an *S. pennellii* introgression is present at the gene’s locus are identified as cis-eQTLs. Example IL chromosomes are represented as rectangles and the gene locus as a star. B, Representative fruit of wild-type (WT), transgenic *SlWD40* RNAi-knockdown and *SlWD40* overexpression lines at several different days post-anthesis (DPA). Figures adapted from Zhu et al. (2022).

Expression-level differences are often mediated by differences in cis-regulatory sequences, such as promoters, which control spatial and temporal gene expression patterns. To find these regions of potential cis-regulation, the authors determined differentially expressed genes in mature fruit in the two species. They then filtered their candidate gene list by performing expression quantitative trait locus (eQTL) analysis on a set of introgression lines (ILs) of *S. pennellii* introgressed into the tomato genome (Eshed and Zamir, 1995). eQTL analyses use mapping populations to determine the genetic loci of expression differences between the introgressed species (Ranjan et al., 2016). Zhu et al. (2022) selected differentially expressed genes whose high expression in tomato fruit decreased specifically in ILs that contained *S. pennellii* genome segments in the candidate gene’s genome position—i.e. these genes’ high expression depended on the cis-regulatory sequence from the tomato genome (Figure 1A). From these analyses they isolated 16 candidate genes for functional validation.

The authors used virus-induced gene silencing to assay the effect of decreasing expression of candidate genes in tomato fruit. Silencing of several candidate genes, including the transcriptional regulator *SlWD40 (WD-40 repeats)*, prevented fruit ripening. *SlWD40* was previously identified as a target of *RIN* (Fujisawa et al., 2013), and Zhu et al. (2022) found *SLWD40* expression increases during fruit maturation similarly to *RIN* expression patterns. The authors generated *SlWD40* transgenic lines, and through visual analysis and quantification of chlorophyll and carotenoid levels they found that overexpression of *SlWD40* accelerated ripening while RNAi-mediated knockdown of *SlWD40* delayed ripening (Figure 1B). They then performed transcriptomic analysis on the transgenic fruit, determining that RNAi-knockdown of *SlWD40* decreased the expression of genes that regulate cell wall modification, ethylene and carotenoid biosynthesis, and sugar metabolism, processes that all promote ripening. This transcriptomic signature was shared with *rin* and *nor* mutants. Through metabolomics assays the authors found that RNAi-knockdown of *SlWD40* expression also decreased the levels of free amino acids in the fruit, which are associated with ripening and the flavor profile of ripe tomatoes, and increased the levels of triacylglycerols, which are broken down during ripening.

Through an innovative, expression-based application of a remarkable historical mapping population, Zhu et al. (2022) elucidated the evolutionarily diverged ripening programs in tomato and its wild relative *S. pennellii*. This work provides insight into key molecular processes associated with desirable fruit crop traits, such as the modification of fruit texture through cell wall remodeling and fruit flavor through drastic global changes in the metabolome of ripening fruit. These traits are appealing targets for engineering food crops (Martín-Pizarro and Posé, 2018). It would be interesting to explore how conserved the master genetic regulators of ripening are in diverged fruit crops which share the fleshy, soft fruit quality of tomatoes. Considering the common shift during domestication from hard, tasteless wild ancestor fruits to the many soft fruit crops consumed today, it may be that selective breeding has acted upon a conserved ripening program despite large evolutionary distances between crop species.

The authors additionally thoroughly characterized a previously uncharacterized regulator of tomato fruit ripening, *SlWD40*. Interestingly, the authors found that RNAi-knockdown of *SlWD40* not only inhibited fruit ripening but also decreased fruit size. Fruit size mostly increases pre-ripening in tomato, and regulators of tomato fruit size are well-characterized targets of selective breeding (Ariizumi et al., 2013). How *SlWD40* interacts with the genetic pathways regulating fruit size is a potential interesting area of future research, especially as the authors observe that auxin homeostasis, known to affect fruit size in tomato, is perturbed in their *SlWD40* transgenic lines.

*SlWD40* was not previously known to regulate ripening, though it had been characterized as a direct target of the master ripening regulator *RIN*. Ripening causes global changes in the transcriptome of fruit, and to what extent *SlWD40* acts as a “master” regulator of this pathway is unclear. Despite being a *RIN* target, *SlWD40* expression patterns closely mimic that of *RIN* and *NOR*, and *SlWD40* knockdown fruit share transcriptomic signatures with *rin* and *nor* mutants. It is unclear whether *SlWD40*’s role in ripening is completely epistatic to *RIN*’s or if there is more complex feedback in this genetic relationship. Generation of higher-order mutants between *SlWD40* and *RIN* and other “master regulators” could establish more clearly the genetics of this pathway. Overall, this work illustrates the complex genetics that underlie this essential transitional state and the agricultural potential of tuning this transition.

*Conflict of interest statement*. The author declares no conflict of interest.
